# Supplementary material for: Carbohydrate Sources Influence the Microbiota and Flavour Profile of a Lupine-Based Moromi Fermentation
Source: Foods. 2023 Jan 2;12(1):197. doi: 10.3390/foods12010197 (PMC9818829; doi:10.3390/foods12010197)
Supplement: Supplementary file 1 [file foods-12-00197-s001.zip › foods-1978790-supplementary.pdf]

Supplementary Data

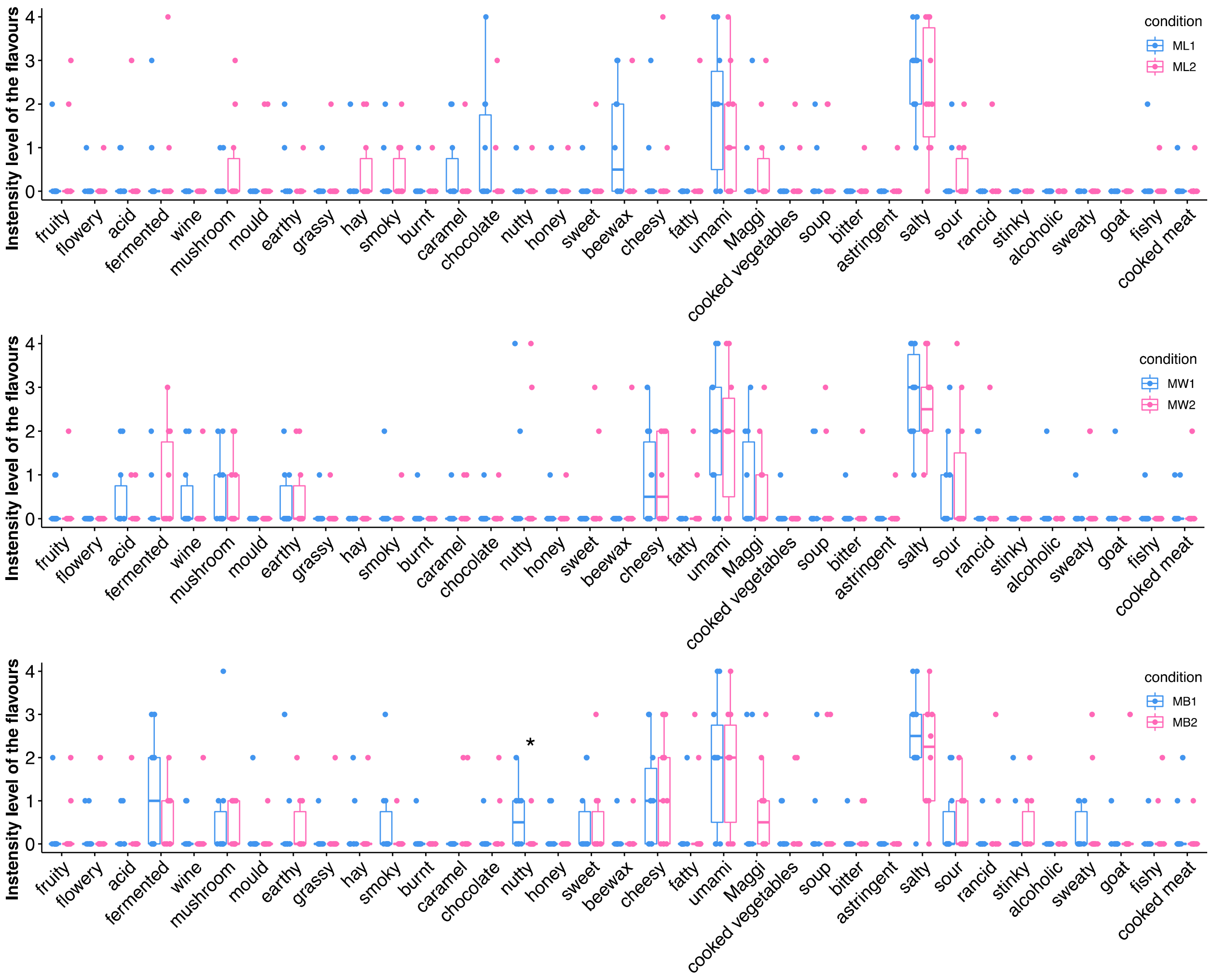

Supplementary Figure S1. Comparison of biological duplicates of Sensory Data. The intensity of aroma and flavour attributes of the replicates of lupine moromi (ML1 and ML2), moromi with wheat (MW1 and MW2), and moromi with buckwheat (MB1 and MB2) are displayed and the difference in the flavour between biological replicates that is rather close to significance is marked with an asterisk (Wilcoxon test, p value=0.059).

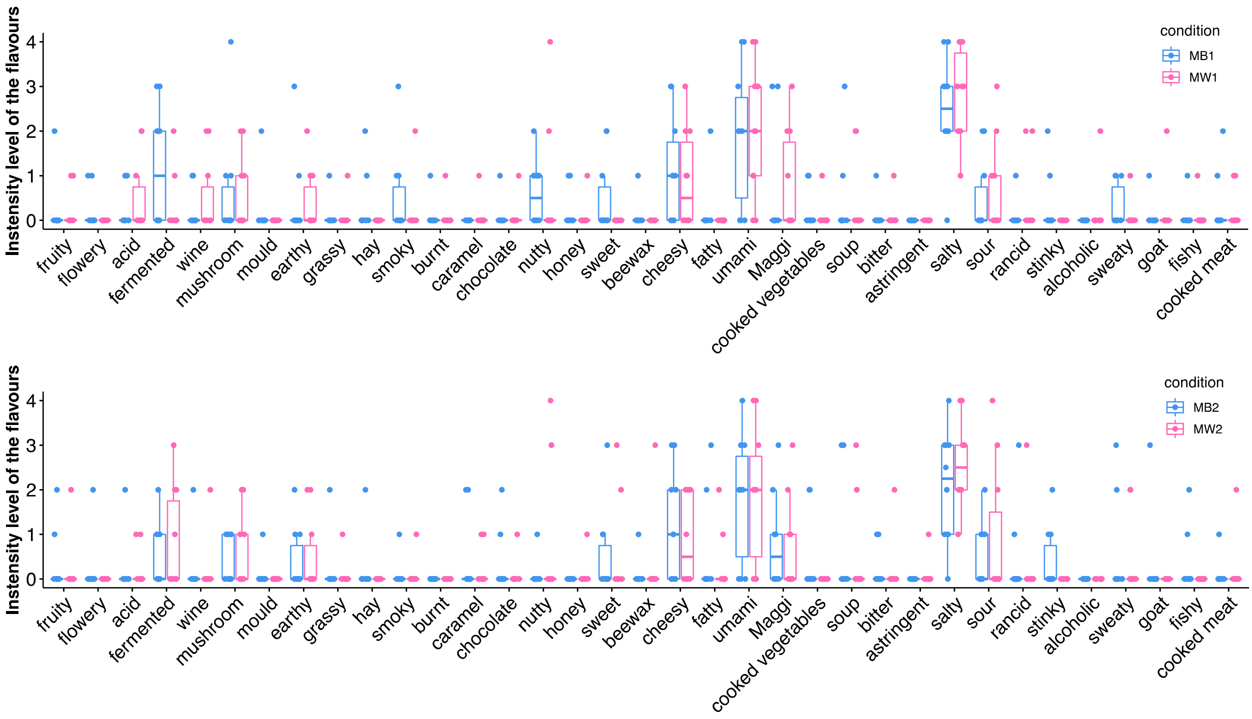

Supplementary Figure S2. Comparison of sensory data of lupine moromi with wheat versus buckwheat. The intensity of aroma and flavour attributes of the replicates of moromi with wheat (MW1 and MW2), and moromi with buckwheat (MB1 and MB2) are displayed and no significant differences were measured.
